# Increased expression of stemness genes *REX-1, OCT-4, NANOG,* and *SOX-2* in women with ovarian endometriosis versus normal endometrium: A case-control study

**DOI:** 10.18502/ijrm.v16i12.3684

**Published:** 2019-01-28

**Authors:** Fatameh Shariati, Raha Favaedi, Fariba Ramazanali, Pegah Ghoraeian, Parvaneh Afsharian, Behrouz Aflatoonian, Reza Aflatoonian, Maryam Shahhoseini

**Affiliations:** ^1^Reproductive Epidemiology Research Center, Royan Institute for Reproductive Biomedicine, ACECR, Tehran, Iran.; ^2^Department of Genetics, Tehran Medical Sciences Branch, Islamic Azad University, Tehran, Iran.; ^3^Department of Genetics, Reproductive Biomedicine Research Center, Royan Institute for Reproductive Biomedicine, ACECR, Tehran, Iran.; ^4^Department of Endocrinology and Female Infertility, Reproductive Biomedicine Research Center, Royan Institute for Reproductive Biomedicine, ACECR, Tehran, Iran.; ^5^Stem Cell Biology Research Center, Yazd Reproductive Sciences Institute, Shahid Sadoughi University of Medical Sciences, Yazd, Iran.

**Keywords:** *Endometriosis*, * Stemness genes.*

## Abstract

**Background:**

Endometriosis is a common, chronic inflammatory disease which is defined as an overgrowth of endometrial tissue outside the uterine cavity. The etiology of this disease is complex and multifactorial but there is a strong evidence that supports the presence of endometrial stem cells and their possible involvement in endometriosis.

**Objective:**

In this study, we analyzed the mRNA expression of *REX-1* stemness gene and reconsidered three other stemness genes *SOX-2, NANOG, OCT-4* in women with endometriosis compared to normal endometrium.

**Materials and Methods:**

Ten ectopic and ten eutopic tissue samples along with 23 normal endometrium specimens were recruited in this study. The expression levels of *OCT-4*, *NANOG*, *SOX-2*, and *REX-1* genes were evaluated by the quantitative real-time polymerase chain reaction.

**Results:**

The transcription levels of *OCT-4*, *NANOG*, and *SOX-2* mRNA were significantly increased in ectopic lesions compared with eutopic and control group (*p* = 0.041, *p* = 0.035, *p* = 0.048), although the *REX-1* mRNA increase was not significant between endometriosis and control groups. Also, there were differences in the expression level of these genes in normal endometrium during the menstrual cycles (*p* = 0.031, *p* = 0.047, *p* = 0.031).

**Conclusion:**

Based on our data, we confirm the dynamic role of stemness genes in proliferation and growth of normal endometrium during the menstrual cycle and conclude that differential expression levels of these genes may contribute to the pathophysiology of endometriosis.

## 1. Introduction

Endometriosis is a gynecological disease in which endometrial glands and stromal tissue grow in areas outside the uterus (1). The multifactorial entity of endometriosis depends on immunologic, hormonal, environmental, and genetic factors. Extensive investigations in recent years lead our mind to the dynamic role of endometrial stem/progenitor cells in the pathogenesis of endometriosis, in the way that `embryonic cell rest theory' has been described as one of the hypotheses for the origin of endometriosis (2).

Based on this theory, many recent studies focused on stemness-related genes, including pluripotency markers such as *Sex determining region Y-box 2 *(*SOX-2*), *NANOG*, and *octamer-binding transcription factor 4 (*OCT-4) (3–5). *SOX-2* regulates different developmental processes via binding to the minor groove of target DNA and play vital and substantial roles in proliferation, differentiation, and fate determination of specific cells (6). *SOX-2* can form a heterodimer with *OCT-4* and regulate self-renewal and pluripotency of embryonic stem cells as well as primordial germ cells (7). Also, *NANOG*, a member of homeobox family genes, has key impress in maintaining of pluripotent stem cells in self-renewal and the undifferentiated conditions (8).

*SOX-2* with *OCT 4* and *NANOG* has been found to cooperatively activate and regulate other stem cells-related gene *REX-1* (Zfp-42) (9). *REX-1* is known as a pluripotency marker usually found in undifferentiated embryonic stem cells (9). It has been declared by investigators that the transcription factors of *OCT 4*, *NANOG, SOX-2*, and *REX-1* are essential elements of self-regulating, and there are strong evidences showing the epigenetic role of these transcription factors in regulation of stem cells (10–13). *OCT-4, SOX-2*, and *NANOG* can induce expression of each other and are necessary to retain the self-renewing and undifferentiated state of blastocyst inner cell mass and embryonic stem cells (8). They also implicate in migration and invasion of cancer cells (5, 8).

On the other hand, *OCT-4* and *SOX-2* form a heterodimer before binding to certain promoters and ripen functionally each other (14). Also, *NANOG* form heterodimer too and make feedback loop to regulate expression of other pluripotency genes (14–17). Among the four aforementioned pluripotency transcription factors, there are some studies that show an aberrant level of *OCT-4, NANOG*, and *SOX-2* in endometriotic tissues (3–5). However, *REX-1* is not identified exclusively in women with ovarian endometriosis in previous studies (3).

As emerging role of stemness genes in the regeneration of endometrium in each cycle, expression profile of four stemness genes (*SOX-2, NANOG, OCT-4*, and *REX-1*) in the normal endometrium of reproductive-aged women during the menstrual cycle were analyzed. Also to investigate the potential role of stemness genes in endometriosis, in this case-control study, the mRNA expression of aforesaid genes in the endometrial tissues of women with endometriosis were compared to normal endometrium.

## 2. Materials and Methods

### Subjects 

Twelve endometriosis women (aged between 20–45 yr) and 23 normal women as control group (aged between 20–45 yr) participated in this case-control study.

All enrolled endometriosis patients were consulting for infertility and/or pelvic pain without hyperplasia or neoplasia. All endometriosis cases were in stages III or IV of disease, based on the revised classification of American Fertility Society (18). Eutopic endometrial biopsies were collected from patients by piple (*n* = 10). Ectopic endometrial tissues were collected from patients with endometrioma who underwent diagnostic laparoscopy for endometriosis (*n* = 10) (Table I). From the total samples of both eutopic (*n* = 10) and ectopic (*n* = 10) endometrial tissues, eight samples were collected from same patients. All the samples were collected through a proliferative phase.

Women of the control group had at least one child by normal gestation with a regular cycle, no evidence of any pathologic uterine disorders and no use of oral contraception or intrauterine device within the last 3 month. Excluding criteria of the control group were endometrial hyperplasia or neoplasia, inflammatory disease or endometriosis in checkup laparoscopy. Endometrial biopsies of this group were obtained during the proliferative phase (*n* = 12) and secretary phase, (*n* = 6) through diagnostic laparoscopic surgery (Table I) and were found not to have endometriosis during surgery. Also, endometrial biopsies in the menses phase were collected from five healthy volunteer women (Table I).

After obtaining tissue samples, every sample was divided into small pieces (50–100 mg) and each piece was placed in RNA later solution straightway and stored in –80${}^{\circ }$C until RNA extraction. For evaluation of the potential role of stemness genes in endometriosis, a study was designed as a case-control study and expression profile of four stemness genes (*SOX-2, NANOG, OCT-4*, and *REX-1*) compared in eutopic, ectopic, and normal endometrial biopsies in the proliferative phase. On the other side to check alteration of stemness genes expression in a menstrual cycle, endometrial biopsies of all control healthy women (Mense phase, *n* = 5, Proliferative phase, *n* = 12, Secretory phase, *n* = 6) were compared together.

### Total RNA extraction and cDNA synthesis

After tissue collection, TRIzol regent was used (Invitrogen, USA) for the extraction of the total RNA from each group according to the manufacturer's instruction and then total RNA was treated with DNase-I endonuclease (EN0521- Thermo Scientific, Germany). cDNA synthesis was performed using RevertAid H Minus First Strand cDNA Synthesis Kit (K1632- Thermo Scientific, Germany) according to the manufacturer's instruction.

### Quantitative real-time PCR 

mRNA expression levels of candidate genes were quantified by real-time PCR (qRT-PCR) as described before (19). Briefly, q-PCR reactions were performed in duplicates on a 7500 Real-Time PCR System (Applied Biosystems, USA) using SYBR Green master mix (Applied Biosystems, USA), with designed primers for *OCT-4, NANOG, SOX-2, REX-1*, and *GAPDH* (*Glyceraldehyde-3-phosphate dehydrogenase)* as an internal control gene (Table II). The specific primers were designed using Primer 3 (version 4.0) (http://primer3.ut.ee/), Gene Runner (version 3.05), and Perl Primer (version v1.1.20) software.

Before order, the primer sequences were checked by Nucleotide Blast and Primer-Blast in the NCBI database and were then purchased from Pishgam Co., Iran. The condition of qRT-PCR reactions was 95${}^{\circ }$C for 10 min, and 40 cycles of 95${}^{\circ }$C for 15 sec and 60${}^{\circ }$C for 60 sec. Gene expression data were analyzed using the ΔΔCt quantitative method to estimate relative fold change values. After each run of PCR, produced melting curve were analyzed for the confirmation of the primers, shown by the presence of a single band and no artifacts of primer-dimer.

All of the samples with a cycle threshold ratio of variation value higher than 1 degree were tested again. For confirmation of melting curve results, agent samples of the real-time PCR products were measured on 2% ultra-pure agarose (Invitrogen, USA, cat no: 16500-100) gel electrophoresis (Payapazhoh pars, Iran), and visualized by ethidium bromide (Sigma Aldrich, USA, cat no: E1510) staining followed by Molecular ImagerⓇ Gel Doc${}^{TM}$ XR+ (BioRad, USA).

### Ethical consideration

This study was approved by the Institutional Ethics Committee of Royan Institute (code: IR.ACECR.ROYAN.REC.1394.77). All participants were aware of the study details, and collection of tissue samples from them were performed after obtaining informed consent with complete verbal and written information.

### Statistical analysis

The results were reported as mean ± SEM. Data were analyzed using One-Way ANOVA followed by Post Hoc Tukey test. The level of *p* < 0.05 was taken as a statistical significance.

## 3. Results

### Comparative evaluation of *OCT-4, NANOG, SOX-2,* and *REX-1* expression in control, eutopic, and ectopic tissues

The mean relative expressions of *OCT-4, NANOG,* and *SOX-2* were significantly higher in ectopic endometrium compared with eutopic and normal endometrium (*p* = 0.041), but the *REX-1* mRNA increase was not significant between endometriosis and control groups. The analysis was carried out on the normal endometrium in the proliferative phase (*p* = 12) and paired eutopic (*p* = 10) and ectopic endometrium specimens (*p* = 10) were in the proliferative phase.

### Expression profile of *OCT-4, NANOG, SOX-2,* and *REX-1* in the control group during the menstrual cycles

**Figure 1:**
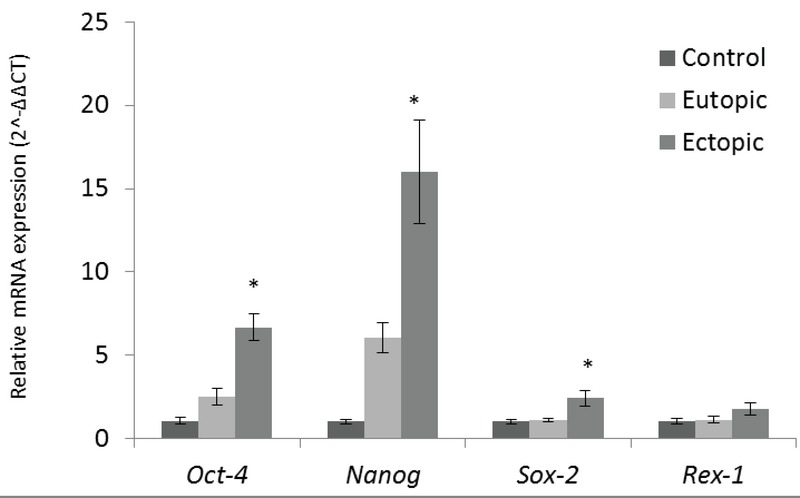
Comparative expression of stemness genes (*SOX-2, NANOG, OCT-4*, and *REX-1*) in proliferative phase in all groups including eutopic and ectopic tissue samples (*n* = 10 in each group) from endometriosis patients as well as normal endometrium samples (*n* = 8) from normal healthy women. The results are expressed as 2${}^{\wedge \Delta \Delta CT}$ (mean ± SEM). Means labeled with asterisks show significant difference versus control group in *p* ≤ 0.05.

**Figure 2:**
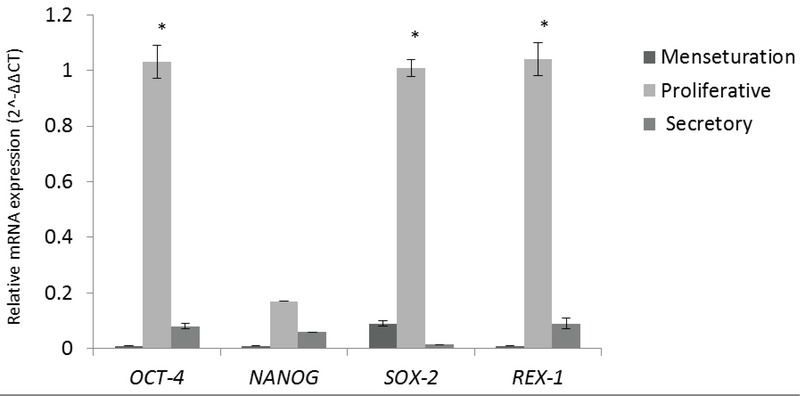
Relative mRNA expression levels of *SOX-2, NANOG, OCT-4,* and *REX-1* stemness marker genes in normal endometrium samples in three phases of the menstrual cycle: menstruation (*n* = 5), proliferative phase (*n* = 12) and secretory phase (*n* = 6). The results are expressed as 2${}^{\wedge }$ΔΔCT (mean ± SEM). Means labeled with asterisks show significant difference versus control group in *p* ≤ 0.05.

Gene expression level of *OCT-4, NANOG, SOX-2*, and *REX-1* were quantitatively evaluated in normal endometrium during the menstrual cycles. *OCT-4, SOX-2*, and *REX-1* showed significantly higher expression in the proliferative phase compared with the secretory phase (*p* = 0.033, *p* = 0.041, *p* = 0.034, respectively, Figure 1). Also, *OCT-4, NANOG*, and *REX-1* showed a significantly higher expression in the proliferative phase compared with the menstrual phase of the cycle (*p* = 0.031, *p* = 0.047, *p* = 0.031, Figure 2).

**Table 1:** Sample groups analyzed in the study.


**Tissue Samples**	**No. of Samples**	**Age (yr) **
Endometriosis (proliferative phase)	12	29.4 ± 1.3
Ectopic	2 + 8*	
Eutopic	2 + 8*	
Control endometrium		
Menstrual phase	5	30.3 ± 1.4
Proliferative phase	12	33 ± 1.7
Secretory phase	6	32 ± 1.15

Note: Data presented as mean ± SD;

*indicates a number of ectopic and eutopic samples obtained from the same patient.

**Table 2:** Primer sequences used in quantitative real-time PCR.


**Gene name**	**Primer sequence 5'→3'**	**Product size**
*GAPDH*	F: CTC ATT TCC TGG TAT GAC AAC GA	122
	R: CTT CCT CTT GTG CTC TTG CT	
*SOX-2*	F: GGG AAA TGG AAG GGG TGC AAA AGA GG	151
	R: TTG CGT GAG TGT GGA TGG GAT TGG TG	
*NANOG*	F: AAA GAA TCT TCA CCT ATG CC	110
	R: GAA GGA AGA GGA GAG ACA GT	
*OCT-4*	F: GTT CTT CAT TCA CTA AGG AAG G	101
	R: CAA GAG CAT CAT TGA ACT TCA C	
*REX-1*	F: TTT ACG TTT GGG AGG AGG	150
	R: GTG GTC AGC TAT TCA GGA G	

Note: GAPDH: Glyceraldehyde-3-phosphate dehydrogenase;

*SOX-2*: Sex determining region Y-box 2;

*NANOG*: Nanog;

*OCT-4*: Octamer-binding protein 4;

*REX-1*: Zfp-42.

## 4. Discussion

Endometriosis is a disease with a complex and multifactorial etiology. Recently, several reports showed that adult progenitor stem cells are responsible for the capacity to generate endometriosis if shed in a retrograde fashion in the menstrual cycle (20). In this regard, there are some evidence showing the functional role of three known stemness markers *OCT-4, NANOG*, and *SOX-2* that synergistically regulate transcription of their target genes in endometrial cells (20–22). Moreover, recent studies have shown a mathematical description of molecular regulation in mouse embryonic stem cell and describe a network structure between *OCT-4, NANOG, SOX-2*, and *REX-1* (21). It is hypothesized that these stemness factors reduce apoptosis, enhance proliferation, higher migration, and invasion ability in the ectopic endometriosis tissue which is migrating to the outside of the uterus (23–25).

In the present study, the mRNA expression level of *NANOG* gene was significantly increased in ectopic endometrial lesions in comparison to eutopic and control groups. This result is similar to previous studies (5, 23), although there is an opposite data showing lower expression of *NANOG* gene in endometriotic tissues by RT-PCR (3). Differential expression of *NANOG* in our study was not observed in eutopic endometrium compared with normal endometrium, the data which was similar to previous reports (23). *OCT-4* gene mRNA expression was increased significantly in ectopic endometrial lesions compared with eutopic and normal endometrium. This expression profile data is in accompany with the previous study that *OCT-4* was up-regulated in ectopic sample tissues (5), but the different expression levels between ectopic endometrium group compared to eutopic and normal endometrium have not been seen in the comparable study (23). The tertiary candidate gene, *SOX-2*, which was checked in this study, showed a significantly higher expression level in ectopic endometrium compared to the eutopic and normal endometrium, consistent with previous reports (3, 4, 23). The last stemness marker gene analyzed through the current study showed no significant differential expression levels between three sample groups (*p* > 0.05). As there are no other quantitative reports about mRNA expression of this gene in endometrial lesions, the current result can be assumed as a minor role of *REX-1* gene compared to *SOX-2, NANOG*, and *OCT-4*, known as central and important factors in regulating the pluripotency properties. However, it should be mentioned that maybe by expanding the study with a higher number of samples, *REX-1* shows a significantly increased expression; the hypothesis needs further experiments.

During this study, parallel to analyzing the endometriotic samples, the expression pattern of the four aforementioned stemness genes was monitored in control endometrium during the menstrual cycle in three phases. Our results showed a significant increase in mRNA levels of *OCT-4, SOX-2,* and *REX-1* in proliferative phase, compared to secretory and menses. About *NANOG*, also an increased expression was observed in the proliferative phase, although it didn't reach a significant level (*p* > 0.05). We can easily construe these results with the dynamic role of stemness genes in the proliferation and self-renewal of normal endometrium.

## 5. Conclusion

Finally, it can be concluded that the altered expression profile of stemness genes in this study indicates more the fundamental role of them in the expatriation, proliferation, invasion, and overall development of endometriosis.

##  Conflict of Interest

The authors declare that they have no conflicts of interest in the research.
